# Extensive horizontal transfer of core genome genes between two *Lactobacillus *species found in the gastrointestinal tract

**DOI:** 10.1186/1471-2148-7-141

**Published:** 2007-08-20

**Authors:** Pierre Nicolas, Philippe Bessières, S Dusko Ehrlich, Emmanuelle Maguin, Maarten van de Guchte

**Affiliations:** 1 INRA, Mathématique Informatique et Génome, UR1077, 78350 Jouy en Josas, France; 2 INRA, Génétique Microbienne, UR895, 78350 Jouy en Josas, France

## Abstract

**Background:**

While genes that are conserved between related bacterial species are usually thought to have evolved along with the species, phylogenetic trees reconstructed for individual genes may contradict this picture and indicate horizontal gene transfer. Individual trees are often not resolved with high confidence, however, and in that case alternative trees are generally not considered as contradicting the species tree, although not confirming it either. Here we conduct an in-depth analysis of 401 protein phylogenetic trees inferred with varying levels of confidence for three lactobacilli from the acidophilus complex. At present the relationship between these bacteria, isolated from environments as diverse as the gastrointestinal tract (*Lactobacillus acidophilus *and *Lactobacillus johnsonii*) and yogurt (*Lactobacillus delbrueckii *ssp. *bulgaricus*), is ambiguous due to contradictory phenotypical and 16S rRNA based classifications.

**Results:**

Among the 401 phylogenetic trees, those that could be reconstructed with high confidence support the 16S-rRNA tree or one alternative topology in an astonishing 3:2 ratio, while the third possible topology is practically absent. Lowering the confidence threshold for trees to be taken into consideration does not significantly affect this ratio, and therefore suggests that gene transfer may have affected as much as 40% of the core genome genes. Gene function bias suggests that the 16S rRNA phylogeny of the acidophilus complex, which indicates that *L. acidophilus *and *L. delbrueckii ssp. bulgaricus *are the closest related of these three species, is correct. A novel approach of comparison of interspecies protein divergence data employed in this study allowed to determine that gene transfer most likely took place between the lineages of the two species found in the gastrointestinal tract.

**Conclusion:**

This case-study reports an unprecedented level of phylogenetic incongruence, presumably resulting from extensive horizontal gene transfer. The data give a first indication of the large extent of gene transfer that may take place in the gastrointestinal tract and its accumulated effect. For future studies, our results should encourage a careful weighing of data on phylogenetic tree topology, confidence and distribution to conclude on the absence or presence and extent of horizontal gene transfer.

## Background

The rapidly growing wealth of genome sequence data from closely related bacteria sheds a new light on bacterial evolution and will become an increasingly important source of knowledge in the near future. Comparative analyses open the way for the identification of factors acting on sequence evolution such as neutral genetic drift, purifying selection or positive selection [1,2]. In addition, comparative studies may use genome data for purposes as diverse as inferring functional links between proteins from phylogenetic occurrence profiles of the proteins [3,4] or searching for functional motifs in non-coding DNA by phylogenetic footprinting [5,6]. These analyses will not reach their full potential, however, without first understanding the genealogical history of the sequences under study. Fundamental questions are: which is the organismal phylogeny? do the histories of individual genes conform to the organismal phylogeny and if not, how do their histories deviate from this phylogeny? can the organismal phylogeny even be recognized? Answers likely depend on the organisms and the evolutionary distances between them. Multi-locus sequence typing data [7,8] show that genetic exchanges can be frequent between strains of a same species and may challenge the idea of reconstructing the organismal phylogeny, defined as the history of the cell lineages across rounds of cell division. At higher phylogenetic levels, the most widely accepted point of view seems to be that a unique tree can still describe the history of a large majority of the conserved sequences, and corresponds to the relationship by descent between the bacterial species [9,10]. However, confidence in phylogenetic tree reconstructions based on individual genes is often weak, and gene transfer can not be excluded [11]. It was also proposed that trees supported by a large number of genes may in some cases reflect preferred routes of gene transfer rather than the lines of descent of the species [12,13]. In this context, we believe that answers could come from more in depth case studies. Here, we present an analysis of the genome history of a particular group of bacteria among the firmicutes that shows intriguing relationships and is of primary importance for humans.

The *Lactobacillus acidophilus *group ("acidophilus complex") is a phylogenetically distinct group of closely related lactobacilli [14], containing species isolated from very diverse environments. Among the best known, *Lactobacillus delbrueckii *ssp. *bulgaricus *(hereafter referred to as *L. delbrueckii*) is extensively used for the production of yogurt and derived dairy products, whereas *Lactobacillus acidophilus *and *Lactobacillus johnsonii *are isolated from the human gut.

Although the contours of this group are clear, the exact relationship between the species within the group is more ambiguous. This ambiguity is illustrated by the fact that the group was originally referred to as the *L. delbrueckii *group [15], a designation which was questioned when it appeared that the GC content of the *L. delbrueckii *genome strongly differed from that of the other genomes in the group. Accordingly, the group was given the name of a more representative member and has since been known as the *L. acidophilus *group [14]. *L. acidophilus *itself has not always been correctly identified. Due to a lack of resolving power of phenotypic classification methods, *L. johnsonii *and *Lactobacillus gasseri *have often been classified as *L. acidophilus *in the past [16].

More detailed knowledge on the phylogenetic relationships within the acidophilus complex will be of particular interest because of the health claims that accompany several species in this group. Yogurt, containing live *L. delbrueckii *and *Streptococcus thermophilus*, has long been recognized for its health benefits and is at the basis of the concept of probiotics [17,18], currently defined by the Food and Agriculture Organization of the United Nations as "live microorganisms which when administered in adequate amounts confer a health benefit on the host". Today, *L. acidophilus *and *L. johnsonii *are both commercialized as probiotics, and are the subject of important research activities aiming to identify the functions and understand the mechanisms involved in probiotic effects. In a more general way, resolution of interspecies relationships of closely related organisms is a prerequisite for an improved comprehension of species evolution and factors contributing in this process.

The genome sequences of *L. johnsonii*, *L. acidophilus *and *L. delbrueckii *have recently become available [19-21] along with the genome sequences of *Lactobacillus plantarum *and *Lactobacillus sakei *[22,23], two more distantly related lactobacilli that do not belong to the acidophilus complex. These data should allow a better understanding of the evolutionary relationship between the lactobacilli of the acidophilus complex. As a first element, van de Guchte *et al*. [21] showed that the similarly sized genomes in this group share a conserved structural backbone, evidenced by a clear global synteny and indicative of their close relatedness. Here we present a more detailed analysis of this relationship using rRNA and protein sequences based phylogeny methods. Our results strongly suggest that extensive horizontal gene transfer took place between the *L. acidophilus *and *L. johnsonii *species lineages, possibly in the gastrointestinal tract.

## Results

### GC content

*L. delbrueckii *has been regarded as standing apart from the other members of the *L. acidophilus *group because of its elevated GC content. The availability of genome sequence and annotation data now allows to study this discrepancy in more detail by a breakdown in GC content at codon positions 1, 2 and 3 in coding sequences (Table 1). The calculated overall GC content of *L. delbrueckii *(49.7%) confirms earlier estimations, largely deviating from the values obtained for *L. acidophilus *and *L. johnsonii *(34.7 and 34.6%, respectively). The difference appears to be much smaller, however, when considering the first codon position only, and almost negligible for the second codon position. In contrast, an extremely high difference is found at the third codon position that shows a 65.0% GC content for *L. delbrueckii *compared to 25.0% and 24.4% for *L. acidophilus *and *L. johnsonii*, respectively.

**Table 1:** GC content by codon position.

	GC	GC1	GC2	GC3
*L. acidophilus*	34.7	46.9	34.0	25.0
*L. johnsonii*	34.6	46.8	33.9	24.4
*L. delbrueckii*	49.7	53.9	36.4	65.0

GC, overall GC content (%); GC1, GC2, GC3, GC content at codon positions 1, 2 and 3, respectively, of coding sequences. For *L. delbrueckii*, which contains an extremely high number of pseudogenes [21], pseudogenes have been excluded from the computation of GC1, GC2 and GC3.

### Protein family occurrence profiles

**Figure 1:**
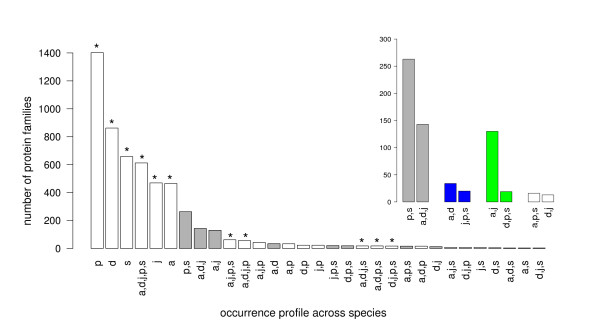
Protein family occurrence profiles. Profiles are listed in decreasing order of abundance. Symbols indicate the species in which the protein families are present: a, *L. acidophilus *(genome size 2.0 Mbp, 1659 protein families); d, *L. delbrueckii *(genome size 1.9 Mbp, 1849 protein families); j, *L. johnsonii *(genome size 2.0 Mbp, 1630 protein families); p, *L. plantarum *(genome size 3.3 Mbp, 2630 protein families); s, *L. sakei *(genome size 1.9 Mbp, 1731 protein families). *, profiles equally compatible with any tree topology. The inset highlights four couples of profiles compatible with the monophyly of the acidophilus complex.: (p.,s.) vs. (a.,d.,j.) equally compatible with any of the three tree topologies depicted in Fig. 2, (a.,d.) vs (j.,p.,s.), (a.,j.) vs. (d.,p.,s.), (d.,j.) vs. (a.,p.,s.) compatible with a single gain or loss event in topology Ta, Tb and Tc, respectively.

A measure of functional similarity between the five lactobacilli considered in this study is obtained from the occurrence profiles of protein families (cf Materials and Methods) across the five species. The results shown in Fig. 1 demonstrate that protein content clearly distinguishes the three lactobacilli of the acidophilus complex from *L. plantarum *and *L. sakei*. After protein families found in only one species and families that exist in all five species, the two most frequent profiles correspond to families with representatives in *L. plantarum *and *L. sakei *but not in the three lactobacilli of the acidophilus complex (263 families), and families that are present in the latter three lactobacilli but not in *L. plantarum *and *L. sakei *(143 families). Within the acidophilus complex, protein content separates *L. delbrueckii *from *L. acidophilus *and *L. johnsonii*. The latter two lactobacilli share 130 families not found elsewhere, a figure that contrasts with the small number of protein families specific to either *L. acidophilus *and *L. delbrueckii *(34 families) or *L. johnsonii *and *L. delbrueckii *(13 families).

### Phylogeny of 16S rRNAs and orthologous proteins

**Figure 2:**
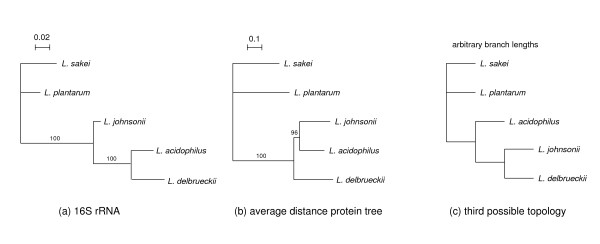
Phylogenetic trees. a, phylogenetic tree based on 16S rRNA sequences by maximum likelihood; b, phylogenetic tree based on average distance from 480 single copy protein families; c, third possible tree topology for the acidophilus complex. Bootstrap support values for internal branches are indicated. Evolutionary distance scales for trees a and b are presented (expected number of substitutions per site). 16S rRNA tree reconstruction is based on a HKY model (expected transition/transversion ratio 1.68 and expected pyrimidine transition/purine transition ratio 0.62) with rate heterogeneity between sites modeled using a gamma distribution with coefficient of variation 2.64. Pairwise evolutionary distances for proteins are estimated with a JTT model and include separate gamma-corrections for each protein family (average coefficient of variation is 1.14).

The evolutionary tree inferred from 16S rRNA sequences is often considered authoritative in bacterial phylogeny reconstruction. In this tree, *L. acidophilus *and *L. delbrueckii *group together while the *L. johnsonii *lineage branches earlier (Fig. 2a). This result may seem surprising, since in apparent contradiction with both the difficulty to distinguish *L. acidophilus *and *L. johnsonii *on the basis of physiological and biochemical properties [16] and the results mentioned above where *L. acidophilus *and *L. johnsonii *look much more alike than *L. acidophilus *and *L. delbrueckii*.

Other powerful approaches to investigate phylogenetic relationships use sequence data of either concatenated proteins [24], or a large number of individual proteins [25,26]. The latter procedure is expected to produce the same tree for the majority of proteins, while a tree with an aberrant branching topology for a particular protein (phylogenetic incongruence) is interpreted as evidence for horizontal gene transfer [27].

A first tree was constructed based on the average distances between species in the 480 single copy protein families (i.e. the protein families that contain exactly one representative in each of the five genomes considered) (Fig. 2b). Surprisingly, the topology of this tree differs from that of the 16S rRNA tree in the branching order of the three species of the acidophilus complex: *L. acidophilus *and *L. johnsonii *group together while the *L. delbrueckii *lineage branches earlier.

Then, trees were reconstructed for individual protein families using the more powerful maximum-likelihood (ML) method. According to this analysis, 401 of the 480 families strongly support the grouping of the three lactobacilli of the acidophilus complex (confidence level ≥ 95% by AU-test). Among these, the relationship between the three members of the acidophilus complex was studied in detail. Of the 79 ML trees that could be reconstructed with ≥ 95% confidence, 47 correspond to the 16S rRNA tree, and 32 to the average protein distance reference tree. No support was found for the third possible topology that groups *L. delbrueckii *and *L. johnsonii *(Fig. 2c). Two aspects of these results are remarkable. First, the 16S rRNA topology (hereafter referred as Ta) and the average protein tree topology (Tb) are found in an astonishing ratio of approximately 3:2. Second, the absence of the third possible topology (Tc) contrasts with the high proportion of proteins supporting the Tb topology.

**Figure 3:**
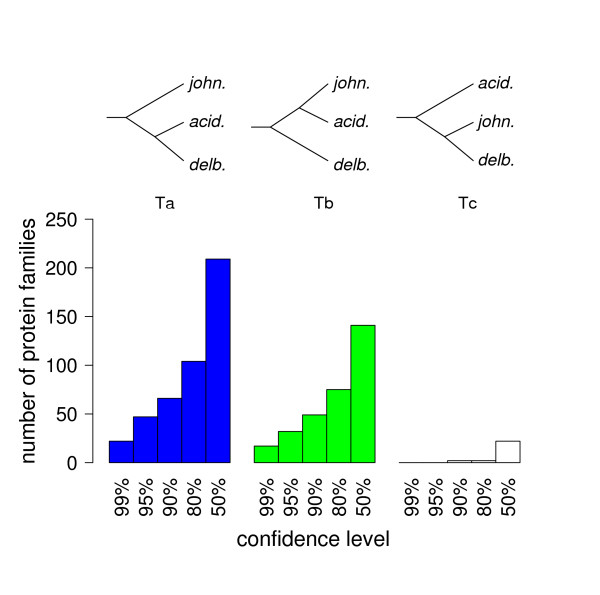
Support for alternative tree topologies. The number of single copy protein families supporting each of the three possible tree topologies for the acidophilus group is indicated as a function of the confidence (by AU test) threshold applied in tree reconstruction for individual families. Only the 401 protein families that strongly support the grouping of the three lactobacilli of the acidophilus complex are represented.

A similar distribution is observed for ML trees at different thresholds of confidence (Fig. 3). Of 181 trees reconstructed with ≥ 80% confidence, 104 correspond to topology Ta, 75 to topology Tb, and 2 to topology Tc. When the confidence threshold is lowered to 50%, 209 proteins are found to support Ta, 141 Tb and 22 Tc. These results are essentially the same as those obtained when only using trees with a confidence level ≥ 95%: a 3:2 ratio between topologies Ta and Tb, and (near) absence of topology Tc. Thus, although individual trees have a slightly higher chance of being erroneous, the overall informational content remains the same. Therefore, proteins with tree confidence levels <95% can be exploited for further analysis, offering the advantages linked to the use of larger datasets.

These results could theoretically have been affected by two well-known sources of artifacts in tree reconstruction. First, incorrect grouping of sequences with similar base composition [28] could have biased tree reconstruction to group *L. acidophilus *and *L. johnsonii *together because of a similar GC content (34.7% and 34.6%, respectively), while diminishing the distance between *L. delbrueckii*, *L. plantarum *and *L. sakei *(49.7%, 45.9% and 41.3% GC, respectively). Although at the protein sequence level this effect would be alleviated, we verified whether *L. delbrueckii *genes supporting topology Ta or Tb (Fig. 3) differed in average GC content. This appeared not to be the case, nor was it the case for the corresponding *L. acidophilus *or *L. johnsonii *genes (see Additional file 1: section 1). As a further check we also reconstructed the gene trees on the basis of nucleotide sequences at the second codon position that shows little differences in GC content between species (Table 1), as suggested by Delsuc, Phillips and Penny [29]. The results are similar to those obtained from the protein sequences, although the confidence in tree reconstruction is generally lower due to the loss of information, and a strong correlation is observed between the protein based trees and the second codon position based trees (see Additional file 1: section 2).

**Figure 4:**
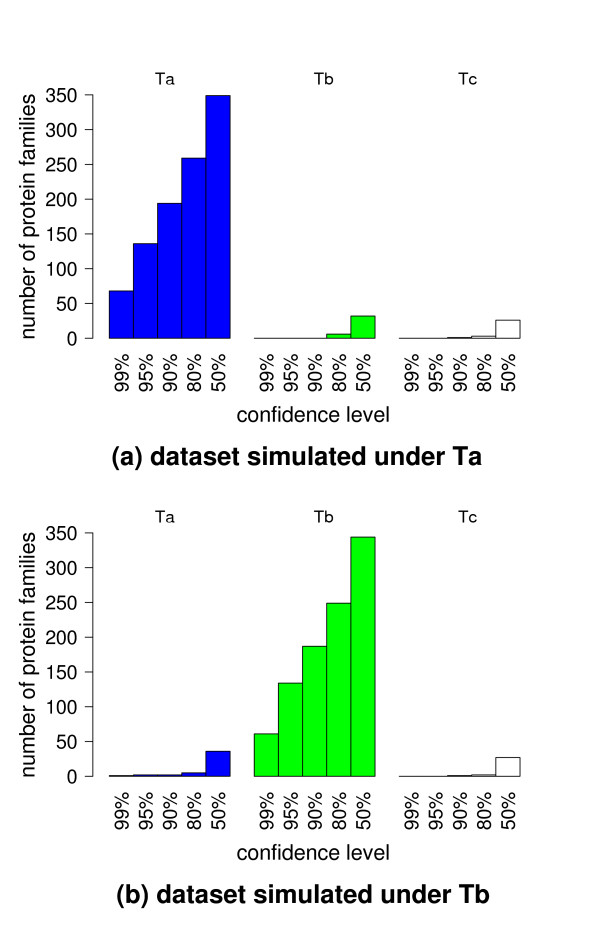
Phylogenetic reconstruction from simulated datasets. Two replicate datasets were simulated under the hypothesis of a unique topology common to all proteins and used in phylogenetic reconstruction. Upper panel: the underlying topology is Ta for all proteins. Lower panel: the underlying topology is Tb. The number of proteins supporting each of the three possible tree topologies Ta, Tb, and Tc (cf Fig. 3) is indicated as a function of the confidence threshold applied in tree reconstruction for individual proteins. Simulations used a JTT model of protein evolution and respected the particular length, site rate heterogeneity and global evolutionary speed of each protein family.

Second, long branch attraction effects (reviewed in [30]) have been described in parsimony reconstruction and may also affect ML reconstruction under some conditions. Therefore, two replicate data-sets were simulated under the hypothesis that either topology Ta or Tb applies to the full set of genes using a parametric bootstrap approach [31]. The results presented in Fig. 4 clearly demonstrate that the distribution pattern observed on the real data set is incompatible with the patterns observed on sequences simulated under a unique topology.

A last argument that seems to rule out the hypothesis of artifacts in tree reconstruction as an explanation for the observation of two major tree topologies is that potential artifacts are unlikely to differentially affect tree reconstruction for different protein families.

Therefore, the highly remarkable fact that two almost equally important evolutionary tree topologies are found instead of one clearly dominant topology strongly suggests that horizontal gene transfer played an important role in the evolution of the three species of the acidophilus complex considered in this study.

### Gene function bias

In order to establish the original species tree we examined whether tree topology was correlated to gene function. Taking the functional classification established for *L. delbrueckii *[21] as a reference (39 categories), a statistically significant overall correlation between topology and functional class was observed when considering protein families for which the tree topology was resolved at a confidence level of 95% or 80%. For classes containing at least 4 proteins with a resolved topology, a Fisher exact test indicates p ~ 0.006 and p ~ 0.027 at the 95% and 80% confidence level, respectively.

This overall correlation is even more significant (p ~ 0.003) for topologies resolved at the 50% confidence level, which still show the 3:2 ratio between topologies Ta and Tb (Fig. 3) and the near absence of topology Tc. In this case, the strongest association between a topology and a particular functional category is found for the category of the aminoacyl-tRNA synthetases that support Tb in a spectacular 15:4 (Tb:Ta) ratio. On the opposite, a large majority of ribosomal proteins (17 versus 6) supports Ta. Other categories supporting preferentially Ta include "membrane bioenergenetics" (8 vs. 0) and the proteins involved in "translation initiation", "translation elongation" and "translation termination" (16 vs. 2). On the basis of current knowledge about gene transfer these results indicate that Ta represents the species tree by descent, and that Tb is the result of horizontal transfer: aminoacyl-tRNA synthetases have often been described as prone to horizontal transfer ([32] and references therein), whereas proteins involved in translation such as ribosomal proteins are generally believed to be refractory to transfer [33].

The bias in gene functions therefore both reinforces the conclusion that the existence of two alternative topologies reflects extensive gene transfer and strongly suggests that the 16S rRNA tree (topology Ta) corresponds to the species relationship by descent.

Remarkably though, a substantial number of genes that are not usually thought of as subject to horizontal transfer support topology Tb at the 80% confidence level (see Additional file 2: Table A2). These include genes involved in fundamental processes as DNA replication (*polA*, *polC*, *dnaE*, *dnaG*), recombination (*recD*, *recJ*, *recN*, *recQ*) and translation (ribosomal protein genes *rpmE*, *rpsJ*; *fusA*, *tsf*), even if the large majority of proteins in these categories support Ta.

### Pairwise evolutionary distances

**Figure 5:**
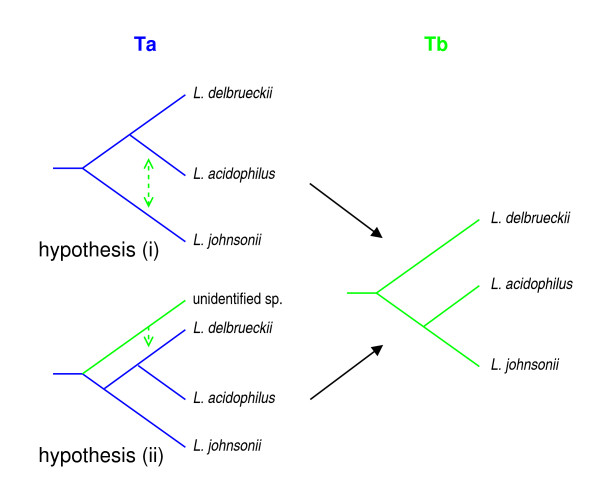
Two hypotheses to explain the coexistence of two tree topologies by horizontal gene transfer. Assuming that Ta represents the original species tree, dashed lines indicate the transfer events that could have generated Tb. Hypothesis (i) corresponds to an exchange between the *L. acidophilus *and *L. johnsonii *lineages. Hypothesis (ii) involves an inflow of genes from an unidentified species in the *L. delbrueckii *lineage. The consequences of these two hypotheses in terms of pairwise evolutionary distances are discussed in the text and compared to the data.

Assuming that gene transfer is at the basis of topology Tb, the question arises whether the lineages concerned by this transfer can be identified. As illustrated in Fig. 5, two hypotheses could explain Tb if Ta reflects the actual species tree: (i) Tb is a consequence of horizontal gene transfer between *L. acidophilus *and *L. johnsonii *or (ii) Tb reflects the horizontal inflow of genes in *L. delbrueckii *from an unidentified species whose lineage branches before the point of divergence of *L. delbrueckii *and *L. johnsonii*. These two hypotheses imply very different predictions about pairwise distances between organisms for proteins supporting either Ta or Tb.

The main prediction of hypothesis (i) is that the distance between *L. acidophilus *and *L. johnsonii *should be smaller for proteins supporting Tb (group B) than for those supporting Ta (group A). Additional consequences for group B proteins may include a smaller distance between *L. johnsonii *and *L. delbrueckii *and a larger distance between *L. acidophilus *and *L. delbrueckii *(see Additional file 3: Fig. A3 and Table A3). The main prediction of hypothesis (ii) is that the distance between *L. acidophilus *and *L. johnsonii *should be the same for group A and group B proteins. Additional consequences for group B proteins may include larger distances between *L. delbrueckii *and *L. johnsonii *as well as between *L. delbrueckii *and *L. acidophilus*.

Comparing evolutionary distances between species for different proteins is complicated by differential protein-specific rates of evolution. This rate is known to vary by more than one order of magnitude between proteins [34,35]. The relative speed of evolution of each protein compared to others has, however, been described as relatively well conserved across lineages [36]. Therefore, to compare pairwise distances between the three lactobacilli of the acidophilus complex for different proteins, the distances were normalized using the distance measured between *L. plantarum *and *L. sakei *for the same protein. The latter distance was verified not to depend on the tree topology (Ta or Tb) supported by the protein (Mann-Whitney test p ~ 0.75).

**Figure 6:**
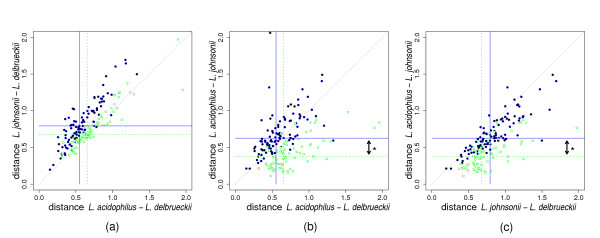
Normalized pairwise distances between the species of the acidophilus complex. Single protein families supporting either topology Ta (closed blue dots) or Tb (open green circles) of Fig. 3 are plotted as a function of normalized pairwise evolutionary distances. Results are presented for protein tree topologies resolved at the 80% confidence level. Horizontal and vertical lines indicate the median of these two groups of proteins on the vertical or horizontal distance axis, respectively (plain blue and dashed green lines for Ta and Tb, respectively). Diagonal dotted lines are drawn to help visualization of the relation between distances on the horizontal and vertical axes. *, the most pronounced difference between Ta and Tb proteins is found in the normalized distances between *L. acidophilus *and *L. johnsonii*.

The 179 protein families of groups A and B for which the trees had been reconstructed with ≥ 80% confidence were then plotted as a function of normalized pairwise evolutionary distances. The results shown in Fig. 6 are clearly in favor of hypothesis (i) and reject hypothesis (ii). The most pronounced difference between groups A and B is found in the normalized distances between *L. acidophilus *and *L. johnsonii*: within the set of 179 proteins, the median is 40% smaller for group B than for group A proteins (Mann-Whitney U-test p ~ 10e-13, Fig. 6bc). In addition, as expected if horizontal transfers occurred relatively recently, group B proteins are concentrated on the equidistance diagonal when plotted as a function of the distance between *L. acidophilus *and *L. delbrueckii *and the distance between *L. johnsonii *and *L. delbrueckii *(Fig. 6a). Our results also suggest that horizontal gene transfer between *L. acidophilus *and *L. johnsonii *took place in both directions as the normalized distances between *L. delbrueckii *and *L. johnsonii *are smaller for group B proteins than for group A proteins, while the inverse is found for the distances between *L. delbrueckii *and *L. acidophilus *(Fig. 6a; see also Additional file 3: Fig. A3 and Table A3).

The results presented in Fig. 6 therefore converge to the conclusion that gene transfer has taken place between *L. acidophilus *and *L. johnsonii*. A detailed analysis of the interspecies distances in Fig. 6 and predictions concerning these distances from 8 different scenarios (see Additional file 3: Fig. A3 and Table A3) provides a second line of evidence to support Ta as the original species tree and Tb as the result of bidirectional horizontal transfer between *L. acidophilus *and *L. johnsonii*.

### Distribution of horizontally transferred genes on the chromosome

To gain more insight into the process of horizontal gene transfer, we examined the relationship between supported phylogeny and chromosomal position of the genes. In particular, we were interested to know whether horizontal transfer events changed the order of the genes on the recipient chromosome and to describe the supported topology in relation to the chromosomal distribution of the genes.

**Figure 7:**
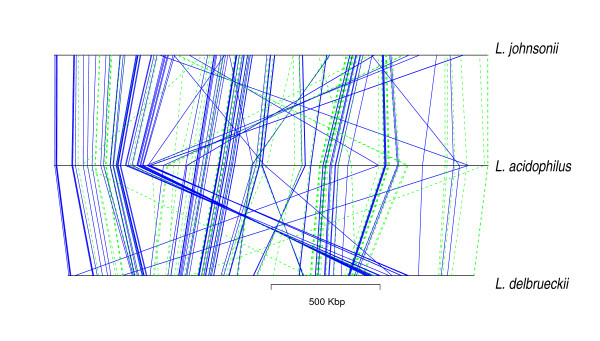
Synteny in the acidophilus complex. Chromosomal positions of the genes whose phylogeny is resolved at the 80% confidence level are indicated. Plain blue and dashed green lines indicate support for topology Ta and Tb, respectively.

Fig. 7 shows the chromosomal positions of the orthologous genes in the three genomes of the acidophilus group together with the topology supported by the corresponding protein families. A global synteny between the three genomes is clearly visible. Horizontal transfer between *L. acidophilus *and *L. johnsonii *did generally not affect synteny. In addition, the limited number of gene order rearrangements that did occur do not preferentially involve genes that support the topology that is characteristic of horizontal gene transfer events (topology Tb).

The alternation of genes supporting either topology strongly suggests that the large number of genes supporting topology Tb were not transferred in a single event, but rather reflect the result of numerous small transfers encompassing no more than a few genes at a time.

## Discussion

In this study, the evolutionary relationship between three lactobacilli of the acidophilus complex, *L. acidophilus*, *L. johnsonii *and *L. delbrueckii*, is clarified. At first sight, *L. acidophilus *and *L. johnsonii *seem the closest related of these three bacteria, sharing the highest number of orthologous genes and a similar GC content largely different from that of *L. delbrueckii*. A similar result was obtained on the basis of fermentation profile grouping [37]. This conclusion is contradicted by 16S rRNA phylogeny, however, which indicates that *L. acidophilus *is more closely related to *L. delbrueckii *than to *L. johnsonii*.

Whole genome sequence information provides two explanations for these paradoxical results. First, the aberrant GC content of *L. delbrueckii *appears to concern GC3 essentially. Furthermore, the *L. delbrueckii *GC3 content sharply deviates from the very strong correlation that is observed between overall GC content and GC3 content in bacteria [21]. As third codon positions are often informationaly neutral in translation and, as a consequence of the resulting lack of selective pressure, evolve more rapidly than first and second codon positions, this suggests that *L. delbrueckii *is in a phase of evolution towards a higher GC content. The different GC content of *L. delbrueckii *thus seems to reflect a recent development, and indicate a different future rather than a different history.

Second, the results of protein phylogenetic analyses suggest that extensive genetic exchange has taken place between the *L. acidophilus *and *L. johnsonii *lineages, giving rise to a closer apparent relationship than expected on the basis of 16S rRNA phylogeny which depicts the historical lines of descent of these species. Very little or no transfer would on the contrary have taken place between *L. delbrueckii *and *L. johnsonii*, while possible transfer between *L. delbrueckii *and *L. acidophilus *could not be detected because this type of transfer would not affect the apparent tree topology of the transferred gene. From the viewpoint of present day knowledge on the habitats of these bacteria these results may not seem surprising as *L. acidophilus *and *L. johnsonii *are found in the same environment, the gastrointestinal tract of humans and animals, while *L. delbrueckii *is found in an isolated environment, yogurt.

It is worth mentioning that in theory topological discordance between gene trees could also result from the coexistence of multiple gene copies along internal branches of the species tree. Differing gene copies may be present within the same genome, as paralogous genes resulting from gene duplication [38], or in different genomes as a consequence of natural intra-species genetic diversity, causing a phenomenon known as incomplete lineage sorting [39,40]. If multiple gene copies were to explain the existence of tree topologies other than that of the species tree (Ta) in the present study, multiple gene copies would need to have existed in the ancestral species lineage and survived until the separation of the *L. acidophilus *and *L. delbrueckii *lineages. In the case of paralogous copies, one copy would subsequently need to have been lost in each of the three terminal lineages as our analysis was restricted to single copy genes. In contrast to horizontal gene transfer, these explanations have in common that gene divergence should always have occurred before species divergence, leading to a longer interspecies distance for the proteins with an alternative tree topology. This does not match with our observation that the pairwise evolutionary distance between *L. acidophilus *and *L. johnsonii *is substantially shorter for those genes that do not support Ta, which instead suggests that the *L. acidophilus *and *L. johnsonii *copies of those genes have a common ancestor in a more recent past than the time of separation of the species lineages.

In addition, these alternative explanations would rather predict the balanced coexistence of Tb and Tc topologies instead of the observed overwhelming preponderance of Tb (see [41] for a detailed mathematical treatment of the possible consequences of incomplete lineage sorting). Finally, the neutral incomplete lineage sorting hypothesis could not explain the observed gene function bias.

Put in a wider perspective, the results presented here show a number of remarkable aspects. The most surprising is the apparent extent of gene transfer events having affected the conserved core genome. Horizontal gene transfer is recognized as a source of the great plasticity of the gene repertoire at all taxonomic levels in the bacterial world [9,13], but is commonly believed to have little or no impact on the genealogy of the conserved core genome. In our data set of 401 conserved protein families (representing about one fifth of the total number of proteins in each of the acidophilus complex species) that fulfilled the requirements to be analyzed, however, protein trees where found to support either the rRNA topology or an alternative topology in an astonishing ratio of about 3:2, independent of the confidence threshold used in tree reconstruction. This result suggests that orthologous gene replacement between two lineages may have affected about 40% of the conserved core genome, a level comparable to that reported between strains of the same species [42]. Although it is true that *L. acidophilus *and *L. johnsonii *are closely related species, their present day DNA sequences are only 51.5% identical, as computed across our 481 alignments. The distribution of transferred genes by *L. acidophilus *– *L. johnsonii *interspecies distance (Fig. 6) suggests that a substantial fraction of the transfer events has taken place relatively recently, at a stage where the DNA sequences of the two species would be expected to already have diverged considerably. To our knowledge, indications for such an important impact of gene transfer on gene genealogies in a group of closely related bacteria were only reported for the Prochlorococcus/marine Synechococcus group of cyanobacteria where genetic exchanges are suspected to have overwhelmed the species genealogy [12].

Our study goes beyond the report of an important level of topological discordance presumed to result from genetic exchanges. The correlation between gene function and topology strengthens the gene transfer hypothesis and makes it possible to disentangle the species genealogy and the gene transfer signal. Examination of the protein divergence data further allowed to locate the transfers between the *L. acidophilus *and *L. johnsonii *lineages yielding a picture coherent with the (current) common ecological niche of these bacteria. A systematic search for gross violations of "clock-like" evolution has been proposed earlier as an efficient screen to detect orthologous gene replacements in large data sets [36]. Here we show that similar ideas can be used to refine the interpretation of topological tree discordance.

The statistically significant correlation between gene function and gene transfer is per-se another important result in the context of orthologous gene replacement between closely related bacterial lineages. It also provides a second indication that a substantial fraction of gene transfer has taken place after *L. acidophilus *and *L. johnsonii *had diverged to a degree that transfer between the two species would not be selectively neutral. Either negative or positive selection could have shape this bias by preventing or facilitating the fixation of transferred alleles. The fact that most of the aminoacyl tRNA-synthetases appear as transferred between *L. acidophilus *and *L. johnsonii *and that some variants of these genes are known to confer antibiotic resistance [32,43] may argue for a role of positive selection. Although the benefit may simply rely in the compensation of deleterious mutations fixed by chance, our results could encourage the search for possible adaptive benefits associated with the other transferred genes.

As indicated above, gene transfer between the *L. acidophilus *and *L. johnsonii *lineages did most likely take place in the gastrointestinal tract, the common habitat of these bacteria. Although several studies demonstrated the transfer of antibiotic resistance genes in the gastrointestinal tract [44], this is to our knowledge the first report to give an indication of the potentially large extent of natural transfer of core genome genes between two bacterial species in this environment and its accumulated effect, a key issue in the context of a growing interest for the metagenome of the human gastrointestinal tract [45].

Finally, our results demonstrate that gene trees even when reconstructed at a relatively low confidence level can be used to reveal horizontal gene transfer. To understand the importance of this statement it is worth comparing our study with those conducted by Daubin, Moran and Ochman [42], Lerat, Daubin and Moran [26] and Lerat *et al*. [9] that used similar tools but concluded that the genealogy of the large majority of orthologous genes conforms to the organismal phylogeny. These authors compared the trees reconstructed from individual genes by evaluating the proportion of genes that shows strong statistical support for a tree differing from a reference tree at a stringent 95% confidence level. This approach would identify only 32 genes as horizontally transferred within the acidophilus complex (i.e. supporting Tb, Fig. 3). However, this does not imply that the large majority of gene genealogies actually conform to the reference tree. In the present study, only 47 of the proteins studied support the 16S rRNA tree (Ta) with ≥ 95% confidence, and thus the astonishing ratio between the two tree topologies remains. The additional information contained in our dataset where comparable proportions of proteins support two tree topologies while the third possible topology is almost absent, independent of the confidence level applied, prompted us thus to adopt another perspective and to reason in terms of support for different topologies, rather than rejection, to determine the extent of horizontal gene transfer. Thus, while our main conclusions are based on phylogenetic tree reconstruction using stringent criteria, the additional information contained in our dataset allowed the use of more data and, therefore, an advanced analysis of our results and extrapolation of our conclusions.

## Conclusion

This case-study reports an unprecedented level of phylogenetic incongruence presumably resulting from extensive horizontal gene transfer between two related bacterial lineages, and gives a first indication of the large extent of gene transfer possibly taking place in the gastrointestinal tract and its accumulated effect. For future studies, our results should encourage a careful weighing of data on phylogenetic tree topology, confidence and distribution to conclude on the absence or presence and extent of horizontal gene transfer. In our particular case, trees resolved with intermediate confidence levels allowed to extrapolate and refine conclusions from high confidence trees on horizontal gene transfer.

## Methods

### Data

This study is based on the five complete *Lactobacillus *genome sequences available by the end of 2005: *L. plantarum *(accession number AL935263 [22]); *L. johnsonii *(AE017198 [19]); *L. acidophilus *(CP000033 [20]); *L. sakei *(CR936503 [23]); *L. delbrueckii *(CR954253 [21]).

### Protein families

Protein families were constructed and only those that contained one representative per species were used for further study. In order to do so, similar proteins were selected on the basis of genome to genome BLASTP comparison with alignments covering at least 80% of both protein sequences and having E-values smaller than 10^-5^. Within this set of proteins, protein families were then defined using the procedure proposed by Lerat, Daubin and Moran [26] where pairs of similar proteins are considered as belonging to the same family when the pairwise similarity level exceeds a certain threshold. Pairwise similarity is measured by the BLAST score ratio (BSR), here computed as the ratio between the raw BLAST score of the pairwise alignment and the maximal raw BLAST score of the alignment of either of the two proteins against itself. Families constructed in this way may contain pairs of genes whose BSR does not exceed the chosen threshold, but which are indirectly connected through pairs that imply other genes. BSR thresholds from 0 to 0.45 were evaluated in steps of 0.05, and the threshold was finally set to 0.30. This choice maximized the number of protein families with exactly one representative in each of the five genomes (480 single copy protein families) and corresponds to the threshold selected by Lerat, Daubin and Moran [26] and Lerat *et al*. [9].

### Phylogenetic analyses

Evolution of the DNA sequences of the small subunit of the ribosome (16S rRNA) and of the proteins of the 480 single copy protein families described above was analyzed using model based approaches. Each set of five sequences was aligned using ClustalW v1.83 [46] with default parameters. Aligned sequences were filtered to remove positions with gaps as well as positions directly adjacent to gaps. This resulted in a 1539 bp long alignment for the 16S rRNA sequences, and a total of 150387 aligned amino-acids for the protein sequences. Parameters of the evolution models for DNA and protein sequences were automatically estimated on each alignment using TREE-PUZZLE [47].

The 16S rRNA alignment served to construct a reference tree by maximum-likelihood (ML) using TREE-PUZZLE with a HKY nucleotide evolution model [48] and a Gamma model [49] for evolution rate variation between sites. Confidence was assessed in 1000 bootstrap replicates generated with the SEQBOOT program of the PHYLIP package v3.6 [50].

A second reference tree was obtained from the protein sequences of the 480 single copy protein families using the neighbor-joining algorithm implemented in the NEIGHBOR program of PHYLIP. For this reconstruction, multiprotein organism pairwise distances were used that account for gene-specific gamma parameters [51]. These distances are obtained as the weighted average (alignments having weights proportional to their lengths) of the pairwise distances computed separately for each protein family using TREE-PUZZLE with a JTT protein evolution model [52]. Confidence was assessed using 1000 bootstrap replicates created by sampling with replacement from the set of 480 alignements.

Phylogenetic reconstructions were also performed separately for each of the 480 protein families using an ML method without molecular clock assumption. For a 5 leaf unrooted tree, 15 topologies are possible which were systematically examined. The site-wise log-likelihoods calculated by TREE-PUZZLE (with option-wsl) for each topology served to evaluate the confidence of the reconstruction using the Approximately-Unbiased test (AU-test) implemented in CONSEL [53,54]. This test computes p-values that delineate embedded confidence subsets of trees among a set of candidates. The confidence level that we report corresponds to one minus the p-value threshold beyond which the confidence subset is not restricted to the single best scoring tree. For those reconstructions that strongly supported the monophyly of the acidophilus complex, a separate AU-test was performed to compare the three possible topologies for the branching of the *L. johnsonii*, *L. acidophilus *and *L. delbrueckii *lineages.

### Parametric bootstrap simulations

Replicate datasets of our 480 protein sequence alignments were simulated with a JTT model using the evolver program included in PAML v3.15 [55] under the hypothesis that the histories of the different genes are all compatible with a unique topology. Branch lengths that served in the simulation were optimized using TREE-PUZZLE for each particular protein family and the simulated data preserved original alignment lengths, amino-acid compositions and variations of the rate of evolution among positions as described by the Gamma parameter. Phylogenetic analyses carried out on replicate datasets conform to the protocol described for the original data.

### Pairwise distances

Pairwise distances between protein sequences were calculated under a JTT model with gamma correction using TREE-PUZZLE.

## Authors' contributions

PN and MvdG designed the study, interpreted the results and wrote the manuscript. PN performed the analyses. PB, SDE and EM contributed to the interpretation and the presentation of the study.

## Supplementary Material

Additional file 1 Controls for possible artifacts in protein phylogeny reconstructions due to GC-content. Provides an analysis of GC-content as a function of supported tree topology, and tree reconstructions based on the DNA sequence at the second codon position. Click here for file

Additional file 2 Horizontally transferred genes. Provides a list of genes that support topology Tb, indicative of horizontal transfer between *L. acidophilus *and *L. johnsonii*. Click here for file

Additional file 3 Horizontal gene transfer scenarios and predicted effects on pairwise evolutionary distances. Provides four hypotheses of horizontal gene transfer to explain the coexistence of two tree topologies, and compares predictions from these hypotheses to observed pairwise evolutionary distances. Click here for file
