# Supplementary material for: Extensive horizontal transfer of core genome genes between two Lactobacillus species found in the gastrointestinal tract
Source: BMC Evol Biol. 2007 Aug 20;7:141. doi: 10.1186/1471-2148-7-141 (PMC1994166; doi:10.1186/1471-2148-7-141)
Supplement: Additional file 1 — Controls for possible artifacts in protein phylogeny reconstructions due to GC-content. Provides an analysis of GC-content as a function of supported tree topology, and tree reconstructions based on the DNA sequence at the second codon position. [file 1471-2148-7-141-S1.pdf]

Controls for possible artifacts in protein phylogeny reconstructions due to GC-content.

1 GC-content as a function of supported tree topology.

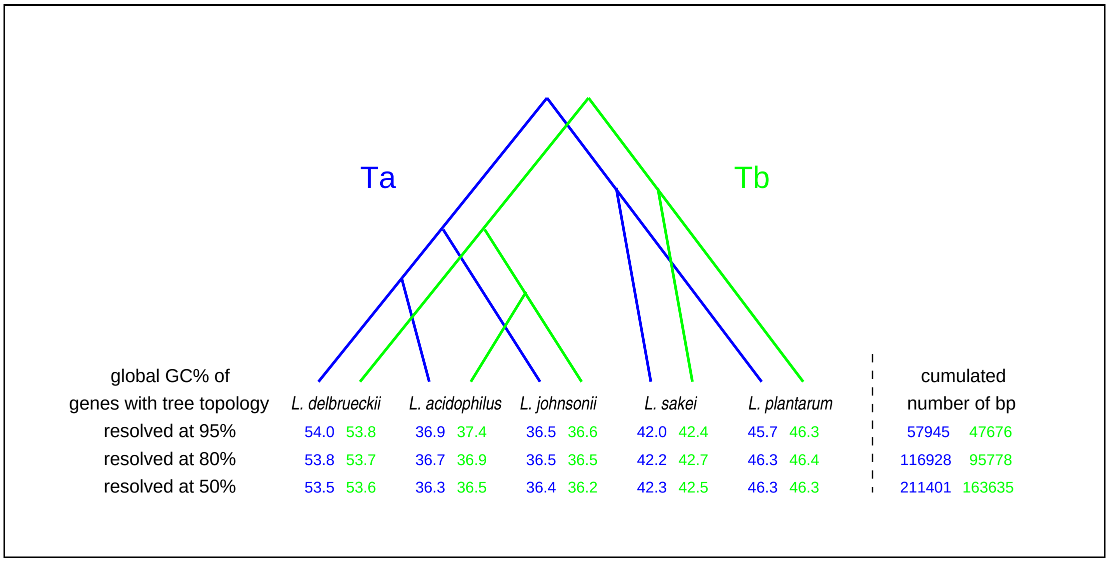

Fig. A1: GC content as a function of supported tree topology. Each of the two main supported topologies is illustrated by a tree (blue for topology Ta, green for topology Tb). GC content computed across the genes that support each topology is shown for each species and for various levels of confidence in tree reconstruction (95%, 80%, 50%). The total numbers of base pairs that served for these computations are reported on the right side of the figure.

The GC-content of the different sets of genes that supported either topology Ta or Tb was examined. As shown in Fig. A1 no differences in GC-content were observed that could explain the support for two different topologies. Within the acidophilus complex, the largest difference in GC-content is found for *L. acidophilus* genes supporting trees resolved with 95% confidence. Within this set of genes, those supporting topology Tb show a slightly higher GC content (37.4 %) than those supporting topology Ta (36.9 %). The p-value for this difference was computed as 0.12 using Pearson's Chi-squared test accounting for the number of base pairs in each set of genes. If an artifact in tree reconstruction due to GC-content existed, a lower GC% for genes supporting Tb than for those supporting Ta would be expected because of the lower overall GC content of *L. acidophilus* and *L. johnsonii* compared to *L. delbrueckii*.

2 Tree reconstructions based on the DNA sequence at the second codon position.

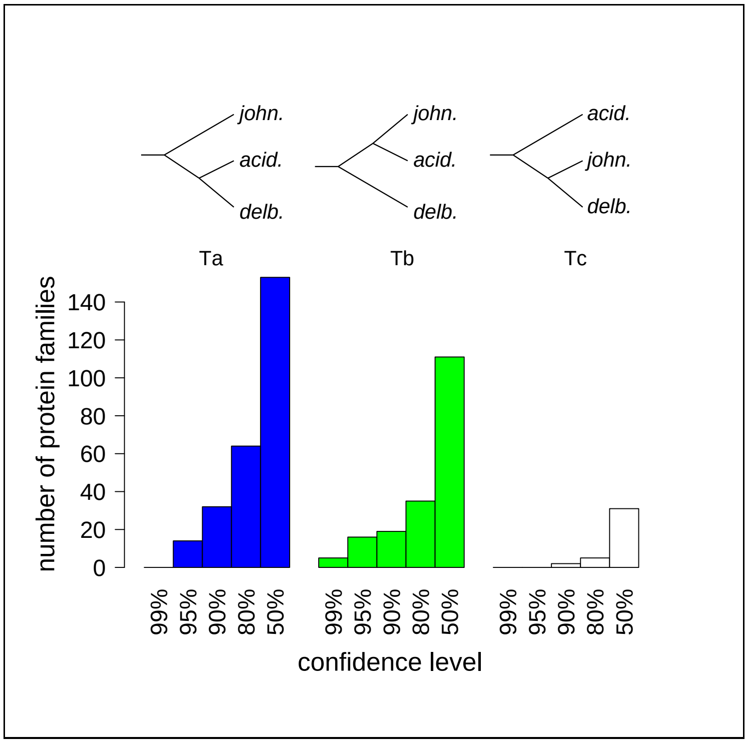

Fig. A2: Second codon position based support for alternative tree topologies. The number of single copy protein families supporting each of the three possible tree topologies for the acidophilus complex is reported as a function of the confidence threshold applied in tree reconstruction.

GC-content at the second position shows little variation among the three lactobacilli of the acidophilus complex. Tree reconstruction based solely on the nucleotides found at the second codon position is therefore expected to be robust against artifacts caused by differences in global GC-content between organisms. We examined trees inferred on the basis of the DNA sequence at the second codon position across alignments derived from protein sequences. ML tree reconstruction was carried out using TREE-PUZZLE with a HKY model of evolution along the same lines as for protein sequences and Figure A2 presents the result in the same format as Figure 3 presents the results for protein ML trees in the body of the manuscript. The picture looks very similar to the one obtained from protein sequence analyses although confidence levels are generally lower (only 31 topologies are resolved at the 95% confidence level compared to 79 when using protein sequences).

confidence threshold	nb topo. resolved	overall concord.	Ta	Tb	Tc
95%	23	100%	12	11	0
80%	75	100%	48	26	1
50%	274	77%	122	79	10

Table A1: Concordance between topologies supported at a same confidence threshold by protein alignment and DNA sequences at the second codon position. Results are shown for three different confidence thresholds. The three rightmost columns report for each topology the number of genes for which both protein and DNA sequence support that topology at the given confidence threshold.

The results presented in Table A1 show the overall agreement between tree topologies inferred from protein and DNA sequences at the second codon position. A 100% match is observed at the 80% confidence level. Genes for which trees inferred from protein and DNA alignments concord support topologies Ta and Tb in the proportion of about 3:2.
