# Supplementary material for: Extensive horizontal transfer of core genome genes between two Lactobacillus species found in the gastrointestinal tract
Source: BMC Evol Biol. 2007 Aug 20;7:141. doi: 10.1186/1471-2148-7-141 (PMC1994166; doi:10.1186/1471-2148-7-141)
Supplement: Additional file 2 — Horizontally transferred genes. Provides a list of genes that support topology Tb, indicative of horizontal transfer between L. acidophilus and L. johnsonii. [file 1471-2148-7-141-S2.pdf]

Table A2. Horizontally transferred genes.

Function	Confidence	Locus tag	Gene	Product
1.1	99.95	Ldb0699	*murE2*	PutativeUDP-N-acetylmuramoylalanyl-D-glutamate-2. 6-diaminopimelate ligase
1.1	99.40	Ldb1522	*murC*	UDP-N-acetylmuramate-alanine ligase
1.1	99.30	Ldb1012	*pbp1A*	Penicillin-binding protein 1A
1.1	97.00	Ldb1542	*pbp2A*	Penicillin-binding protein 2A
1.2	100.00	Ldb1353	-	ABC transporter. ATP-binding/permease protein
1.2.4	94.80	Ldb0577	*ptsI*	Phosphoenolpyruvate-protein phosphotransferase (enzyme I of the phosphotransferase system)
1.2.4	94.20	Ldb2040	-	PTS system. fructose-specific enzyme IIABC component
1.2.5	97.80	Ldb1299	-	Amino Acid ABC transporter. substrate binding /permease protein
1.2.6	100.00	Ldb0545	*pyrP*	Uracil permease
1.2.6	85.40	Ldb0295	*pbuX*	Xanthine permease
1.7	81.60	Ldb0846	*scpB*	Putative segregation and condensation protein B
2.1.1	87.60	Ldb0531	*rpiA*	Ribose 5-phosphate isomerase
2.1.2	99.80	Ldb0636	*pgk*	Phosphoglycerate kinase
2.2	99.70	Ldb1575	*metK*	S-adenosylmethionine synthetase
2.2	98.60	Ldb0494	*glmS*	Glutamine-fructose-6-phosphate transaminase (isomerizing)
2.2	95.90	Ldb0360	*alr*	Alanine racemase
2.2	88.00	Ldb1472	*glnA*	Glutamine synthetase (Glutamate-ammonia ligase)
2.3	99.70	Ldb1531	*pyrF*	Orotidine-5'-phosphate decarboxylase
2.3	99.70	Ldb0298	*guaA*	GMP synthase
2.3	97.40	Ldb1532	*pyrE*	Orotate phosphoribosyltransferase
2.3	95.50	Ldb0296	*xpt*	Xanthine phosphoribosyltransferase
2.3	90.60	Ldb0291	*purA*	Adenylosuccinate synthetase
2.3	90.30	Ldb0354	*pyrG*	CTP synthase
2.3	86.30	Ldb1308	*apt*	Adenine phosphoribosyltransferase
2.4	82.30	Ldb1347	-	1-acyl-sn-glycerol-3-phosphate acyltransferase
2.5	92.70	Ldb0728	*folC1*	Folylpolyglutamate synthase
2.5	89.30	Ldb0725	*thiI*	Thiamine biosynthesis protein ThiI
2.5	86.80	Ldb1510	*coaE*	Dephospho-CoA kinase
3.1	99.90	Ldb1512	*polA*	DNA polymerase I
3.1	92.70	Ldb1247	*dnaG*	DNA primase
3.1	90.70	Ldb1337	*polC*	DNA polymerase III. alpha subunit (gram-positive type) (PolC)
3.1	86.80	Ldb0837	*dnaE*	DNA polymerase III. alpha subunit (DnaE)
3.2	86.40	Ldb0170	*mpg*	3-methyladenine DNA glycosylase
3.3	99.99	Ldb0758	*recD*	Exodeoxyribonuclease V alpha chain
3.3	97.90	Ldb0814	*recQ*	ATP-dependent DNA helicase RecQ
3.3	93.50	Ldb1420	*recN*	RecN. ATPase involved in DNA repair
3.3	91.30	Ldb1309	*recJ*	RecJ. single-stranded DNA specific exonuclease
3.3	83.30	Ldb1511	*fpg*	DNA-formamidopyrimidine glycosylase
3.3	82.80	Ldb1006	*nth*	Endonuclease III
3.5.2	93.80	Ldb0658	-	Putative transcriptional regulator (MarR family)
3.6	99.90	Ldb0591	-	rRNA methylase
3.6	95.10	Ldb1280	*cca*	tRNA nucleotidyltransferase
3.6	92.30	Ldb1489	-	tRNA/rRNA methyltransferase
3.6	88.80	Ldb1380	*rnc*	Ribonuclease III
3.6	85.10	Ldb2215	*trmE*	tRNA modification GTPase trmE
3.7.1	94.10	Ldb0356	*rpmE*	50S ribosomal protein L31
3.7.1	83.60	Ldb0395	*rpsJ*	30S ribosomal protein S10
3.7.2	99.50	Ldb0727	*valS*	Valyl-tRNA synthetase
3.7.2	99.10	Ldb0256	*tyrS*	Tyrosyl-tRNA synthetase
3.7.2	98.80	Ldb1570	*leuS*	Leucyl-tRNA synthetase
3.7.2	96.80	Ldb0889	*aspS*	Aspartyl-tRNA synthetase

3.7.2	95.80	Ldb0469	*gatA*	Glutamyl-tRNA(Gln) amidotransferase subunit A
3.7.2	95.50	Ldb1486	*pheT*	Phenylalanyl-tRNA synthetase. beta subunit
3.7.2	94.10	Ldb0373	*lysS*	Lysyl-tRNA synthetase
3.7.2	87.90	Ldb1506	*thrS*	Threonyl-tRNA synthetase
3.7.2	87.20	Ldb1338	*proS*	Prolyl-tRNA synthetase
3.7.4	98.60	Ldb0394	*fusA*	Translation elongation factor G (EF-G)
3.7.4	98.20	Ldb1344	*tsf*	Translation elongation factor Ts
3.8	85.40	Ldb1310	*srtA*	Sortase
3.8	83.60	Ldb1104	*ppiB*	peptidyl-prolyl cis-trans isomerase (rotamase).cyclophilin type
3.8	81.10	Ldb0702	*hemK*	Methylase of polypeptide chain release factors
3.10	99.97	Ldb2034	*pepF*	Oligoendopeptidase F
3.10	94.40	Ldb1621	*gcp*	Putative glycoprotein endopeptidase
3.10	86.50	Ldb1339	-	Putative metalloprotease
4.1	99.60	Ldb1313	*dnaK*	Chaperone protein DnaK (heat shock protein)
5.2	99.00	Ldb1637	-	Putative methyltransferase
5.2	98.70	Ldb2051	-	GTP-binding protein
5.2	94.60	Ldb0272	-	Putative hydrolase (HD superfamily)
5.2	93.60	Ldb2100	-	Putative glutamine amidotransferase
5.2	90.40	Ldb0622	-	Conserved hypothetical protein
5.2	89.20	Ldb1509	-	Conserved hypothetical protein
5.2	89.20	Ldb0883	-	Conserved hypothetical protein
5.2	86.90	Ldb0465	*pcrA*	ATP-dependent DNA helicase PcrA
5.2	83.80	Ldb2216	-	Conserved hypothetical membrane protein
5.2	82.00	Ldb0766	-	Putative methylase

Genes are listed that support topology Tb, indicative of horizontal transfer between *L. acidophilus and L. johnsonii*. Function: 1.1, Cell wall; 1.2, Transport/binding proteins and lipoproteins; 1.2.4, Transport/binding of carbohydrates; 1.2.5, Transport/binding of amino-acids; 1.2.6, Transport/binding of nucleosides, nucleotides, purines and pyrimidines; 1.7, Cell division; 2.1.1, Specific carbohydrate metabolic pathway; 2.1.2, Main glycolytic pathways; 2.2, Metabolism of amino acids and related molecules; 2.3, Metabolism of nucleotides and nucleic acids; 2.4, Metabolism of lipids; 2.5, Metabolism of coenzymes and prosthetic groups; 3.1, DNA replication; 3.2, DNA restriction and modification (and repair); 3.3, DNA recombination, and repair; 3.5.2, Transcription regulation; 3.6, RNA modification; 3.7.1, Ribosomal proteins; 3.7.2, Aminoacyl-tRNA synthetases; 3.7.4, Translation elongation; 3.8, Protein modification; 3.10, Protein degradation; 4.1, Adaptation to atypical conditions; 5.2, Protein of unknown function similar to proteins from other organisms. Confidence, confidence in tree reconstruction (%) computed by AU-test. Locus tag, gene identifier in the *L. delbrueckii* ATCC11842 genome (van de Guchte et al., 2006. Proc. Natl. Acad. Sci. USA. 103:9274-9279.).
