# Supplementary material for: Extensive horizontal transfer of core genome genes between two Lactobacillus species found in the gastrointestinal tract
Source: BMC Evol Biol. 2007 Aug 20;7:141. doi: 10.1186/1471-2148-7-141 (PMC1994166; doi:10.1186/1471-2148-7-141)
Supplement: Additional file 3 — Horizontal gene transfer scenarios and predicted effects on pairwise evolutionary distances. Provides four hypotheses of horizontal gene transfer to explain the coexistence of two tree topologies, and compares predictions from these hypotheses to observed pairwise evolutionary distances. [file 1471-2148-7-141-S3.pdf]

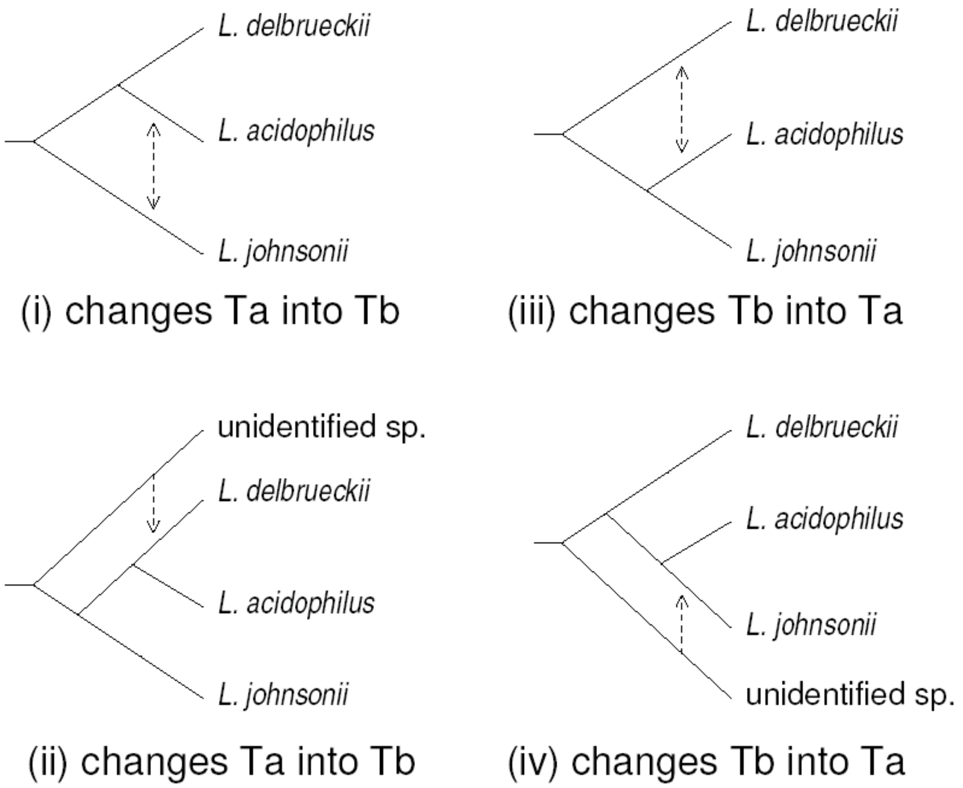

Fig. A3. Four hypotheses of horizontal gene transfer to explain the coexistence of two tree topologies. Dashed lines indicate horizontal gene transfer.

Theoretically, four hypotheses can explain the coexistence of two tree topologies by horizontal gene transfer. Hypotheses (i) and (ii) presume that the 16S rRNA tree (Ta) represents the original species tree, and explain the occurrence of the alternative tree Tb by horizontal gene transfer. Hypotheses (iii) and (iv) presume that Tb represents the original species tree. As explained in the text, when taking the observed correlation between protein functional class and supported tree topology into account only hypotheses (i) and (ii) should be considered, and among these hypothesis (i) with a scenario of bidirectional gene transfer fits all data. Table A3 shows that the same conclusion is reached when comparing predictions from all four hypotheses above to the observed pairwise interspecies distances presented in Fig. 6.

Table A3. Horizontal gene transfer and predicted effects on pairwise evolutionary distances.

	distance<br>*L. acidophilus*<br>*L. delbrueckii*	distance<br>*L. acidophilus*<br>*L. johnsonii*	distance<br>*L. bulgaricus*<br>*L. johnsoni*
hypothesis I predictions			
a) transfer from *L. acidophilus* to *L. johnsonii*	dTb = dTa	dTb < dTa	dTb < dTa
b) transfer from *L. johnsonii* to *L. acidophilus*	dTb > dTa	dTb < dTa	dTb = dTa
c) transfer in both directions	dTb > dTa	dTb < dTa	dTb < dTa
hypothesis II predictions			
transfer from unidentified to *L. delbrueckii*	dTb > Ta	dTb = dTa	dTb > dTa
hypothesis III predictions			
a) transfer from *L. acidophilus* to *L. delbrueckii*	dTb > Ta	dTb = dTa	dTb > dTa
b) transfer from *L. delbrueckii* to *L. acidophilus*	dTb > Ta	dTb < dTa	dTb = dTa
c) transfer in both directions	dTb > Ta	dTb < dTa	dTb > dTa
hypothesis IV predictions			
transfer from unidentified to *L. johnsonii*	dTb = Ta	dTb < dTa	dTb < dTa
observations (cf Fig. 6)	dTb > Ta	dTb << dTa	dTb < dTa

The predicted effect of the different horizontal gene transfer scenarios depicted in Fig. A3 on pairwise evolutionary distances is indicated. dTa, dTb: average pairwise distance for proteins supporting topology Ta or Tb (Fig. 2), respectively. Predictions are only qualitative. No predictions are made on the extent of the differences that may be expected. Predictions highlighted in red are incompatible with the observations from Fig. 6. The observations from Fig. 6 support scenario Ic: topology Ta corresponds to the original species tree, and topology Tb is the result of bidirectional horizontal gene transfer between *L. acidophilus* and *L. johnsonii.*
